# Our Journal

**Published:** 1859-12

**Authors:** 


### Our Journal.

The cause of the delay in the issue of the Journal, was explained in connection with the last number, (the office and fixtures of the Publisher being consumed by fire, with the loss of the November number which was just ready for issue,) and is now operating in preventing the regular issue of the Journal. We hope, however, by the last of February to have both the January and February numbers issued, after which time, our usual regularity will be observed. This difficulty has been entirely beyond our control, and every effort has been made to meet it in the best manner practicable.

